# Squinted Airborne Synthetic Aperture Radar Imaging with Unknown Curved Trajectory

**DOI:** 10.3390/s20216026

**Published:** 2020-10-23

**Authors:** Wei Pu, Junjie Wu, Yulin Huang, Jianyu Yang

**Affiliations:** 1 Department of Electronic and Electrical Engineering, University College London, London WC1E 6BT, UK; 2 School of Information and Communication Engineering, University of Electronic Science and Technology of China, 2006 Xiyuan Road, Gaoxin Western District, Chengdu 611731, China; junjie_wu@uestc.edu.cn (J.W.); yulinhuang@uestc.edu.cn (Y.H.); jyyang@uestc.edu.cn (J.Y.)

**Keywords:** highly squinted synthetic aperture radar (SAR), non-linear chirp scaling (NLCS), motion modelling and optimization, differential evolution (DE)

## Abstract

The imagery of airborne highly squinted synthetic aperture radar (SAR) with curved trajectory is a challenging task due to the translational-variant range cell migration (RCM) and azimuth modulation. However, in most cases of practical application, the curved trajectory cannot be accurately known, which brings greater difficulties to the imaging problem. To accommodate these issues, we propose a novel motion modelling and optimisation based imaging algorithm for the highly squinted SAR with unknown curved trajectory. First, to correct the translational-variant RCM, a coarse-to-fine RCM correction scheme as well as a range perturbation approach is applied. Afterwards, an optimisation model of motion information under the criterion of minimum entropy is built during the azimuth processing by nonlinear chirp scaling (NLCS). Correspondingly, a differential evolution (DE) optimisation strategy is proposed to estimate the motion information in an iterative manner. We empirically compare the proposed algorithms with several state-of-the-art highly squinted curved SAR imaging algorithms. Numerical results show the effectiveness of the proposed method in the case without any prior information of the curved trajectory.

## 1. Introduction

**Figure 1:**
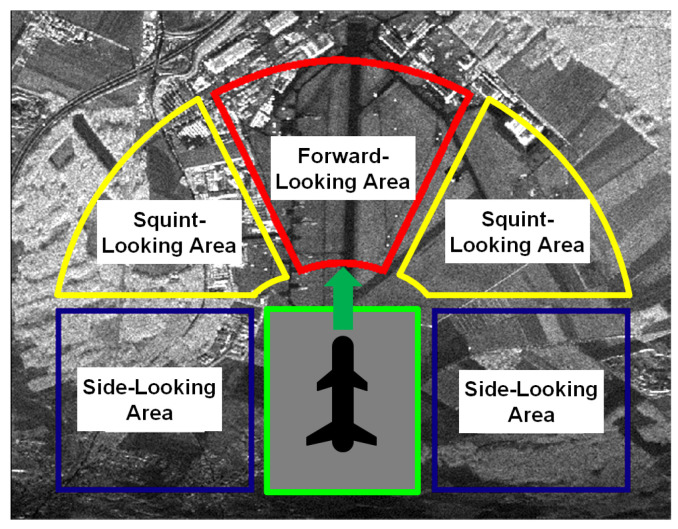
Different synthetic aperture radar (SAR) systems.

Synthetic aperture radar (SAR) [1,2,3] finds wide applications in both civilian and military domains due to its capability of operating under any weather conditions and any time of the day. SAR is an active radar with its own illumination to image a ground scene of interest with high resolution. Many types of improved SAR systems have emerged in recent years [4,5,6]. According to the angle between the radiation direction of the radar and movement direction, SAR systems can be divided into three categories: (1) side-looking SAR, (2) squint-looking SAR and (3) forward-looking or back-looking SAR, as shown in Figure 1. Among them, due to its manoeuvrability and flexibility, squinted SAR is a research hotpot [7,8,9,10]. In a conventional squinted SAR system, the platform moves along a straight trajectory. However, in many new applications, more and more SAR systems are mounted on the high-speed and manoeuvrable platform—such as unmanned aerial vehicles, air fighters, missile, satellite and so on—and the radar platform moves along a curved trajectory [11,12,13,14]. In this paper, we focus on the airborne squinted SAR system.

Squinted SAR imaging with known curved trajectory—where we have the the prior information of the moving curved trajectory of the radar platform—has been studied in various literature, and can be categorised into two groups:

- *Time-domain imaging algorithm:* The time-domain imaging algorithm—also known as Back Projection (BP) algorithm—is viewed as the ideal solution to the focusing problem; however, it is also time-consuming. There are many modifications to BP, and among them, the most popular one is fast factorised BP (FFBP) [15,16,17,18,19,20]. In a recursive manner, the computation cost is greatly reduced by FFBP and also could be further accelerated by parallel processing. Another advantage of time-domain imaging algorithms is that they are adaptable, and there is no need for significant modification to applying conventional time-domain imaging algorithms—where platform moves along a linear trajectory—to the squinted SAR imaging with curved trajectory [21,22].
- *Frequency-domain imaging algorithm:* Some frequency domain algorithms—including chirp scaling (CS) [23], nonlinear chirp scaling (NLCS) [24,25], Omega-K and their extensions [12,26]—are proposed to achieve well-focused image for squinted SAR with curved trajectory. These frequency-domain algorithms are well-designed to cope with the transnational variant range cell migration (RCM) and azimuth phase in a perturbation, equalisation or interpolation manner.

However, in practical application, there is always some motion uncertainty—which means the deviations of the actual trajectory from the pre-designed one—for the moving platform, mainly caused by air turbulence in airborne SAR system. As a consequence, the focusing quality of the existing imaging algorithms mentioned above is strongly impaired. Principally speaking, we can get the motion information of the moving platform through orbit and altitude data provided by an ancillary instrument such as inertial measurement units (IMU), inertial navigation system (INS) and global positioning system (GPS) [27,28,29,30,31]. However, unfortunately, measurement uncertainties on the IMU, INS, GPS and so on would limit the accuracy of the moving information, and the measurement remains unknown in many real applications including some unmanned aerial vehicles without an ancillary instrument. Thus, imaging techniques of squinted SAR without any prior information of the curved moving trajectory are of great value.

Note that this problem is similar with the autofocusing problem [32,33,34] in SAR motion compensation, as both of them focus on eliminating the uncertainty of the platform’s motion that deviates from the expected trajectory. However, these two types of problems are not completely consistent, i.e., they and their corresponding solutions differ in the aims and constraints. The differences lie in the following factors.

- *Purpose:* The imaging and autofocusing are an organic combination to achieve high-precision imaging together. The imaging algorithms aim to focusing SAR echo under several geometry parameters—motion parameters like initial position, speed and acceleration of the platform. Comparatively, the purpose of the autofocusing algorithms is to compensate for the motion errors—caused by unexpected deviation from the geometry configuration—which are not taken into consideration in the imaging algorithms.
- *Constraint:* The imaging algorithms cannot deal with the high frequency motion uncertainty as the they are parametrically designed for the geometry parameters. The constraint for the autofocusing algorithms is that they cannot compensate for the motion uncertainty with large amplitude. The corresponding reason is that the complex motion errors—that are always with high-order terms or even non-parameterisable—lead to many assumptions including approximation on residual range cell migration (RCM), neglecting translational variance on azimuth modulation.

Consequently, the existing autofocusing methods cannot solve the imaging problem without any prior information of the curved trajectory.

To accommodate for this problem, a novel squinted SAR imaging algorithm without any prior information of the curved trajectory is proposed. The main contributions are as follows.

1. To correct the translational variant RCM, a coarse-to-fine RCM correction scheme integrated with a range perturbation approach is proposed. The coarse-to-fine RCM correction scheme works in an iterative manner, which could guarantee the prerequisite of the following processors on azimuth modulation. A range perturbation approach is utilised to correct the translational variant RCM based on the estimated motion parameters.
2. We establish an optimisation model for the motion parameters under the minimum entropy criterion based on NLCS processing. At this stage, the translational variance of the azimuth phase modulation has been taken into consideration and the problem is changed into a multi-variable minimisation problem.
3. The minimisation problem is solved by a differential evolution (DE) strategy. The estimated motion parameters would be utilised to guide the RCM correction in the next iteration, which results in a more accurate estimation iteratively.
4. We conduct experiments using synthetic SAR data to show the effectiveness of the proposed method.

The rest of this paper is organised as follows. Section 2 introduces squinted SAR with curved trajectory. In Section 3, a motion modelling and optimisation-based imaging algorithm is derived in detail. We make some comments on the proposed method in Section 4, and numerical results are given in Section 5. Section 6 concludes this paper.

## 2. Squinted Sar with Curved Trajectory

Assuming that the transmitted signal is p(τ), the SAR echo corresponding to arbitrary target P(x,y) the imaging scene could be formulated as

(1) $$\begin{array}{c} s\left(\tau ,t\right)=\omega_{a}\left(t\right)\exp\left(-j4\pi f_{c}\frac{r\left(t;x,y\right)}{c}\right)p\left(\tau -\frac{2r\left(t;x,y\right)}{c}\right), \end{array}$$

where τ and *t* denote the fast and slow time, respectively, while *x* and *y* represent the azimuth and range directions with respect to imaging scene σ, respectively. In (1), $\omega_{a}$ is the azimuth envelope, r(x,y,n) the range history, $f_{c}$ the carrier frequency and *c* the signal speed. After range compression, SAR echo is changed into

(2) $$\begin{array}{c} s_{r}\left(\tau ,t\right)=\omega_{a}\left(t\right)\exp\left(-j4\pi f_{c}\frac{r\left(t;x,y\right)}{c}\right)\mathrm{sinc}\left(\tau -\frac{2r\left(t;x,y\right)}{c}\right), \end{array}$$

where sinc() represents the range envelope after range compression.

**Figure 2:**
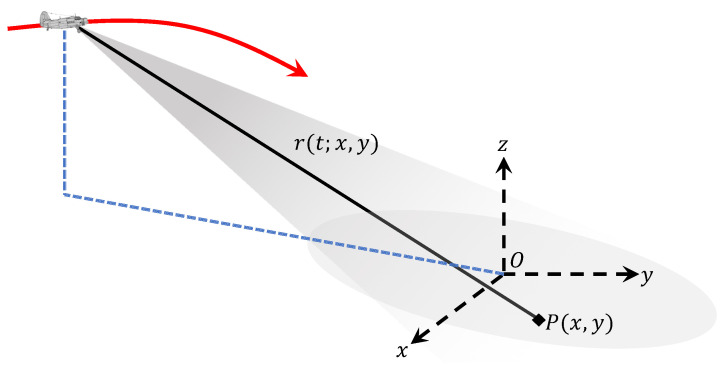
Geometry configuration of highly squinted SAR with curved trajectory.

Figure 2 presents the geometry configuration of the squinted airborne SAR with motion errors. Assume that the radar platform moves along the direction of *y* axis with a constant velocity *v* as well as accelerations $a_{x}$ and $a_{z}$ along *x*- and *z*-axes, and the cases with more complex motions will be discussed in Section 4. Note that higher-order motion along *y*-axis is originated from along-track nominal velocity changes, and it is generally compensated via azimuth resampling of SAR raw data [7,35]. Consequently, higher-order motion along *y*-axis is not taken into consideration in this paper. *x* is the ground direction orthogonal to *y*. We choose the centre point of the imaging scene, i.e., target *O* in Figure 2, as the coordinate origin. *t* denotes the azimuth time, and it is chosen to be zero at the composite beam centre crossing time of target *O*.

Suppose target P(x,y) is an arbitrary target located at the imaging area, and the corresponding range history of *P* can be formulated as

(3) $$\begin{array}{c} r\left(t;x,y\right)=\sqrt{{\left(x_{0}+a_{x}t^{2}-x\right)}^{2}+{\left(y_{0}+vt-y\right)}^{2}+{\left(z_{0}+a_{y}t^{2}\right)}^{2}}, \end{array}$$

where $x_{0}$, $y_{0}$ and $z_{0}$ denote the initial position coordinate of the radar platform with respect to *x*, *y* and *z* directions, respectively. $a_{x}$ and $a_{z}$ denote the accelerations along *x* and *z* directions, respectively.

In order to facilitate subsequent imaging processing, a third-order polynomial range model is utilised to accurately characterise r(t;x,y) as

(4) $$\begin{array}{c} r\left(t;x,y\right)=k_{0}+k_{1}\left(t-\frac{y}{v}\right)+k_{2}{\left(t-\frac{y}{v}\right)}^{2}+k_{3}{\left(t-\frac{y}{v}\right)}^{3}+\ldots \end{array}$$

where

(5) $$\begin{array}{c} k_{0}=\sqrt{{\left(x_{0}+\frac{a_{x}y^{2}}{v^{2}}-x\right)}^{2}+y_{0}^{2}+{\left(z_{0}+\frac{a_{x}y^{2}}{v^{2}}\right)}^{2}}, \end{array}$$

(6) $$\begin{array}{cc} k_{1} & ={\left.\frac{\partial r(t;x,y)}{\partial t}\right|}_{t=y/v}\\ \; & =\frac{\left(4a_{x}y\left(-x+x_{0}+\frac{a_{x}y^{2}}{v^{2}}\right)\right)/v+2vy_{0}+\left(4a_{z}y\left(\frac{a_{z}y^{2}}{v^{2}}+z_{0}\right)\right)/v}{2{\left({\left(-x+x_{0}+\frac{a_{x}y^{2}}{v^{2}}\right)}^{2}+y_{0}^{2}+{\left(\frac{a_{z}y^{2}}{v^{2}}+z_{0}\right)}^{2}\right)}^{0.5}}, \end{array}$$

and

(7) $$\begin{array}{cc} k_{2}= & {\left.\frac{\partial^{2}r\left(t;x,y\right)}{2\partial t^{2}}\right|}_{t=y/v}\\ = & \frac{v^{4}+\left(6a_{x}^{2}+6a_{z}^{2}\right)y^{2}+v^{2}\left(-2a_{x}x+2a_{x}x_{0}+2a_{z}z_{0}\right)}{2v^{2}{\left({\left(-x+x_{0}+\frac{a_{x}y^{2}}{v^{2}}\right)}^{2}+y_{0}^{2}+{\left(\frac{a_{z}y^{2}}{v^{2}}+z_{0}\right)}^{2}\right)}^{0.5}}\\ \; & -\frac{{\left(\left(2a_{x}y\left(-x+x_{0}+\frac{a_{x}y^{2}}{v^{2}}\right)\right)/v+vy_{0}+\left(2a_{z}y\left(a_{z}y^{2}+v^{2}z_{0}\right)\right)/v^{3}\right)}^{2}}{4{\left({\left(-x+x_{0}+\frac{a_{x}y^{2}}{v^{2}}\right)}^{2}+y_{0}^{2}+{\left(\frac{a_{z}y^{2}}{v^{2}}+z_{0}\right)}^{2}\right)}^{1.5}} \end{array}$$

In (4), $k_{3}$ is the third-order term of the polynomial; however, we do not show the concrete expression of $k_{3}$ which would not be used in the following.

## 3. Motion Modelling and Optimisation

Many literatures are focused on the curved trajectory squinted SAR imaging problem; however, all of them need to know the accurate information of motion parameters. Once there is a slight error occurring in these parameters, the corresponding imaging quality is greatly reduced, which will be illustrated in the Section 5. In this paper, our goal is to develop a imaging algorithm not needing the prior information of the motion parameters, which could achieve a well-focused SAR image as well as estimate the motion parameters at the same time.

In order to solve the squinted SAR imaging problem with unknown curved trajectory, there are three issues to be solved: (1) translational variant RCM correction, (2) translational variant azimuth modulation and (3) unknown motion parameters. Two solutions are proposed to solve these issues: (1) equalise and correct the translational variant RCM by range perturbation processing, and (2) model and estimate the motion parameters through a NLCS processing with azimuth variance of the Doppler parameters taken into consideration, which solve the last two issues at the same time. However, there is a contradiction between the two solutions—the RCM should be corrected to ensure an accurate NLCS-based motion estimation, while an accurate RCM correction need the estimated motion parameters. Here, we apply a coarse-to-fine RCM correction scheme to address this contradiction.

### 3.1. Coarse-to-Fine Rcm Correction Scheme

The coarse-to-fine RCM correction scheme begins with the observation that if the the residual RCM after RCM correction is smaller than one range resolution, the motion estimation can be implemented correctly. The correction scheme works in an iterative manner.

**Figure 3:**
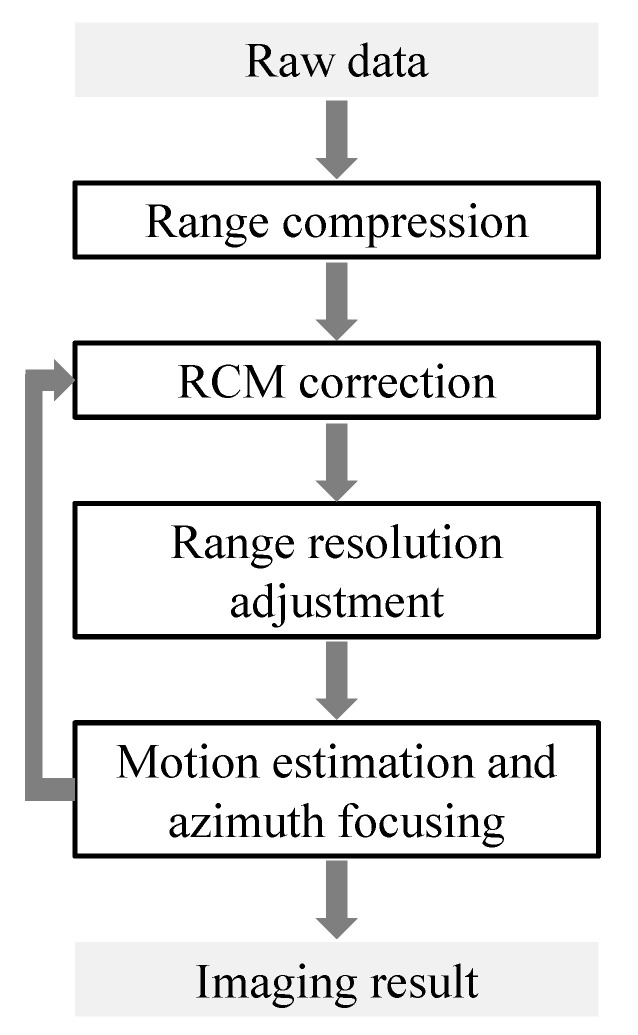
Block diagram of the coarse-to-fine range cell migration (RCM) correction scheme.

At first, we use the autofocus RCM correction algorithm in [36] to compensate for the translational-invariant component of the RCM, and the translational variant part remains to be corrected as it is larger than one range resolution in most cases. Then, the echo is changed into a coarser range resolution by adjusting the bandwidth so that the uncompensated RCM is within one range resolution. Once done, the motion parameters can be accurately estimated and azimuth focusing can be implemented from the SAR data with coarser resolution through a NLCS processing. Using estimated and updated motion parameters, the RCM is compensated by a range perturbation processing with translational variance taken into consideration. Then, we adjust range resolution into a finer one, and implement a more accurate motion parameter estimation. The block diagram of the coarse-to-fine RCM correction scheme is shown in Figure 3.

Note that the RCM correction in the first iteration is implemented using the RCM estimation and correction algorithm in [36], and that in the following iterations is performed by range perturbation processing.

The proposed algorithm stops until the change rate of the estimation of motion parameters is smaller than a predetermined threshold. The change rate in the k+1th iteration is defined as

(8) $$\begin{array}{c} c^{k+1}=\frac{|a_{x}^{k+1}-a_{x}^{k}\left|+\right|a_{z}^{k+1}-a_{z}^{k}|}{|a_{x}^{k}\left|+\right|a_{z}^{k}|}, \end{array}$$

where $a_{x}^{k}$ and $a_{z}^{k}$ represent the estimation of $a_{x}$ and $a_{z}$ at the *k*th iteration.

### 3.2. RCM Correction

In the first iteration, we do not have any estimation on the motion parameters $a_{x}$ and $a_{z}$, and therefore the RCM is corrected in an autofocus manner [36,37]. In this algorithm, the RCM of the most dominant target can be estimated and compensated without needing the motion information; however, the translational-variance of RCM is neglected.

In the following iterations, the RCM will be compensated more accurately based on the estimation of $a_{x}$ and $a_{z}$ from the last step “Motion estimation and azimuth focusing” shown in Figure 3. In the squinted SAR, the range migration is dominated by linear range walk, and a linear range cell migration correction (LRCMC) should be employed first. However, it introduces the azimuth-variance with respect to both RCM and azimuth modulation. Here, a translational-variant RCM correction processing based on range perturbation [23] is utilised as follows.

1. Multiplying LRCMC factor $H_{rcm1}\left(t,f_{\tau }\right)$ with signal $s_{1}\left(t,f_{\tau }\right)$ in the range frequency and azimuth time domain to correct the linear range walk, where $H_{rcm1}\left(t,f_{\tau }\right)$ is
  (9) $$\begin{array}{c} H_{rcm1}\left(t,f_{\tau }\right)=\exp\left(j\frac{4\pi (f_{c}+f_{\tau })t}{c}k_{10}\right), \end{array}$$
  $f_{\tau }$ denotes the range frequency, $s_{1}\left(t,f_{\tau }\right)$ is obtained by applying Fourier transform in τ dimension for data $s_{1}\left(t,\tau \right)$ in (2) and
  (10) $$\begin{array}{c} k_{10}=k_{1}{|}_{x=0,y=0}=\frac{vy_{0}}{{\left(x_{0}^{2}+y_{0}^{2}+z_{0}^{2}\right)}^{0.5}}. \end{array}$$
2. Multiplying range perturbation factor $H_{1}\left(f_{t},f_{\tau }\right)$ with signal obtained by step 1 in 2-D frequency domain to equalise the translational variance of range curvature introduced by LRCMC, where
  (11) $$\begin{array}{c} H_{1}\left(f_{t},f_{\tau }\right)=\exp\left(j\pi \frac{c^{2}f_{\tau }f_{t}^{3}}{2{\left(f_{c}+f_{\tau }\right)}^{3}}\left(p_{1}+\frac{p_{2}c}{2(f_{c}+f_{\tau })}f_{t}\right)\right). \end{array}$$
  In (11), $f_{t}$ denotes the azimuth frequency, and $p_{1}$ and $p_{2}$ are
  (12) $$\begin{array}{c} p_{1}=-\frac{k_{11}^{2}k_{20}^{2}+4k_{10}k_{11}k_{20}k_{21}+k_{10}^{2}k_{21}^{2}}{12k_{10}k_{20}^{7}k_{11}^{4}\left(3k_{10}k_{21}+2k_{11}k_{20}\right)}, \end{array}$$
  (13) $$\begin{array}{c} p_{2}=-\frac{2k_{11}k_{20}k_{21}+k_{10}k_{21}^{2}}{12k_{10}k_{20}^{7}k_{11}^{4}\left(3k_{10}k_{21}+2k_{11}k_{20}\right)}, \end{array}$$
  where
  (14) $$\begin{array}{c} k_{11}={\left.\frac{\partial k_{1}}{\partial y}\right|}_{x=0,y=0}=\frac{2(a_{x}x_{0}+a_{z}z_{0})}{v\sqrt{x_{0}^{2}+y_{0}^{2}+z_{0}^{2}}}, \end{array}$$
  (15) $$\begin{array}{cc} k_{20}=k_{2}{|}_{x=0,y=0}= & \frac{v^{2}+2a_{x}x_{0}+2a_{z}z_{0}}{\sqrt{x_{0}^{2}+y_{0}^{2}+z_{0}^{2}}}\\ \; & -\frac{v^{2}y_{0}^{2}}{{\left(x_{0}^{2}+y_{0}^{2}+z_{0}^{2}\right)}^{1.5}}, \end{array}$$
  and
  (16) $$\begin{array}{c} k_{21}={\left.\frac{\partial k_{2}}{\partial y}\right|}_{x=0,y=0}=-\frac{4y_{0}\left(a_{x}x_{0}+a_{z}z_{0}\right)}{{\left(x_{0}^{2}+y_{0}^{2}+z_{0}^{2}\right)}^{1.5}}. \end{array}$$
  After the range perturbation processing, the range curvatures for different targets in a given range bin are uniform and can be compensated simultaneously.
3. Multiplying factor $H_{2}\left(f_{t},f_{\tau }\right)$ with signal obtained by step 2 to compensate for the range curvature correction (RCC) and second compression correction (SRC) in 2-D frequency domain, where
  (17) $$\begin{array}{c} H_{2}\left(f_{t},f_{\tau }\right)=\exp\left(-j\pi (\Phi_{rcc}\left(f_{t}\right)f_{\tau }+\Phi_{src}\left(f_{t}\right)f_{\tau }^{2})\right). \end{array}$$
  $\Phi_{rcc}\left(f_{t}\right)$ and $\Phi_{src}\left(f_{t}\right)$ stand for the correction phases for RCC and SRC, respectively, where
  (18) $$\begin{array}{cc} \Phi_{rcc}\left(f_{t}\right)= & \frac{{2r\left(t;x,y\right)|}_{t=0,x=0,y=0}}{c}-\frac{k_{10}^{2}}{2ck_{2}}-\frac{k_{10}^{3}k_{30}}{4ck_{20}^{3}}\\ \; & +\frac{cf_{t}^{2}}{8k_{20}f_{c}^{2}}+\frac{3\lambda k_{10}k_{30}f_{t}^{2}}{16k_{20}^{3}f_{c}}+\frac{\lambda^{2}k_{30}f_{t}^{3}}{16k_{20}^{3}f_{c}}, \end{array}$$
  and
  (19) $$\begin{array}{c} \Phi_{src}\left(f_{t}\right)=\frac{3\lambda^{2}k_{30}f_{t}^{3}}{16k_{20}^{3}f_{c}^{2}}-\frac{cf_{t}^{2}}{4k_{20}f_{c}^{3}}+\frac{3\lambda k_{10}k_{30}f_{t}^{2}}{8k_{20}^{3}f_{c}^{2}}. \end{array}$$
  In (18) and (19),
  (20) $$\begin{array}{c} {r\left(t;x,y\right)|}_{t=0,x=0,y=0}=\sqrt{x_{0}^{2}+y_{0}^{2}+z_{0}^{2}}. \end{array}$$
  In addition, $k_{20}$ and $k_{30}$ are given by (21) and (22), respectively.
  (21) $$\begin{array}{cc} k_{20}=k_{2}{|}_{x=0,y=0} & ={\left.\frac{\partial^{2}r\left(t;x,y\right)}{2\partial t^{2}}\right|}_{t=y/v,x=0,y=0}\\ \; & =\frac{v^{2}+2a_{x}x_{0}+2a_{z}z_{0}}{2\sqrt{x_{0}^{2}+y_{0}^{2}+z_{0}^{2}}}-\frac{v^{2}y_{0}^{2}}{4{\left(x_{0}^{2}+y_{0}^{2}+z_{0}^{2}\right)}^{1.5}} \end{array}$$
  (22) $$\begin{array}{cc} k_{30}=k_{3}{|}_{x=0,y=0} & ={\left.\frac{\partial^{3}r\left(t;x,y\right)}{6\partial t^{3}}\right|}_{t=y/v,x=0,y=0}\\ \; & =\frac{v^{3}y_{0}^{3}}{2{\left(x_{0}^{2}+y_{0}^{2}+z_{0}^{2}\right)}^{2.5}}-\frac{vy_{0}\left(v^{2}+2a_{x}x_{0}+2a_{z}z_{0}\right)}{2{\left(x_{0}^{2}+y_{0}^{2}+z_{0}^{2}\right)}^{1.5}} \end{array}$$
4. The high-order term of RCM is compensated by
  (23) $$\begin{array}{c} H_{rcm2}\left(t,f_{\tau }\right)=\exp\left(j\frac{4\pi (f_{c}+f_{\tau })}{c}r_{h}\left(t\right)\right), \end{array}$$
  where
  (24) $$\begin{array}{c} r_{h}{\left(t\right)=r\left(t\right)|}_{x=0,y=0}-k_{10}t-k_{20}t^{2} \end{array}$$
  is the high-order term of RCM.

### 3.3. Range Resolution Adjustment

In subsection B, most but not all of the RCM has been corrected. To deal with the residual RCM, we manage to adjust the range resolution to a coarse one so that the residual RCM is within one range resolution. With iterative RCM correction scheme proceeding, the estimation of $a_{x}$ and $a_{z}$ become more and more accurate, and the coarse resolution can be adjusted to a finer one gradually [38,39]. The coarse-to-fine range resolution can be implemented as

(25) $$\begin{array}{c} \rho_{r}^{k+1}=\rho_{r}\left(1+ab^{k}\right), \end{array}$$

where a>1 is a constant, which stands for the range resolution amplification factor; b<1 is the changing rate of range resolution; the range resolution of the *k*th iteration is denoted by $\rho_{r}^{k}$; and $\rho_{r}$ is the range resolution of the original SAR data. Here, we choose a=10 and b=0.4 in this paper. Due to the fact that the range resolution is linear to the inverse of the bandwidth $B_{r}$, as

(26) $$\begin{array}{c} \rho_{r}=\frac{c}{2B_{r}}, \end{array}$$

the range resolution adjustment can be implemented through changing the bandwidth of the range signal. Specifically speaking, the bandwidth $B_{r}$ can be adjust by filtering the SAR data in the range frequency domain. At the k+1th iteration, the frequency filtering width is set to be

(27) $$\begin{array}{c} B_{r}^{k+1}=\frac{B_{r}}{1+ab^{k}}. \end{array}$$

### 3.4. Motion Modelling Based on Nlcs

As analysed in [40], the targets in different range bin—with the same ${r\left(t;x,y\right)|}_{t=0}$—will be relocated into the same range gate after LRCMC, which introduce azimuth-variance with respect to Doppler parameters. For the purpose of evaluating the azimuth variance of the Doppler parameters, the relationship between coordinates *x* and *y* in a certain range bin is given by

(28) $$\begin{array}{c} r_{0}=\sqrt{{\left(x-x_{0}\right)}^{2}+{\left(y-y_{0}\right)}^{2}+z_{0}^{2}}, \end{array}$$

where $r_{0}$ is the slant range for the given range bin. The corresponding meaningful solution for (28) is

(29) $$\begin{array}{c} x=f\left(r_{0},y\right)=x_{0}-\sqrt{r_{0}^{2}-{\left(y-y_{0}\right)}^{2}-z_{0}^{2}}, \end{array}$$

To modulate the azimuth-variant Doppler parameters and focus the curved trajectory squinted SAR echo, we model the Doppler centroid $f_{dc}$ and Doppler FM rate $f_{dr}$ as

(30) $$\begin{array}{c} f_{dc}=f_{dc0}+f_{dc1}y, \end{array}$$

and

(31) $$\begin{array}{c} f_{dr}=f_{dr0}+f_{dr1}y+f_{dr2}y^{2}. \end{array}$$

According to the range history defined in (3) and its polynomial model, the parameters in (30) and (31) can be obtained by

(32) $$\begin{array}{c} \left\{\begin{array}{c} f_{dc0}=\left(-2k_{1}/\lambda \right){|}_{x=f(r_{0},y),y=0}\\ f_{dr0}=\left(-2k_{2}/\lambda \right){|}_{x=f(r_{0},y),y=0}\\ f_{dc1}=\left(-2\partial k_{1}\left(x,y\right)/\left(\lambda \partial y\right)\right){|}_{x=f(r_{0},y),y=0}\\ f_{dr1}=\left(-2\partial k_{2}\left(x,y\right)/\left(\lambda \partial y\right)\right){|}_{x=f(r_{0},y),y=0}\\ f_{dr2}=\left(-\partial^{2}k_{2}\left(x,y\right)/\left(\lambda \partial y^{2}\right)\right){|}_{x=f(r_{0},y),y=0} \end{array}\right., \end{array}$$

where

(33) $$\begin{array}{c} k_{1}{|}_{x=f(r_{0},y),y=0}=\frac{vy_{0}}{r_{0}}, \end{array}$$

and

(34) $$\begin{array}{cc} \; & k_{2}{|}_{x=f(r_{0},y),y=0}\\ \; & =\frac{v^{2}+2a_{x}\sqrt{r_{0}^{2}-y_{0}^{2}-z_{0}^{2}}+2a_{z}z_{0}}{r_{0}}-\frac{v^{2}y_{0}^{2}}{r_{0}^{3}}. \end{array}$$

In addition, the partial derivatives of $k_{1}\left(x,y\right)$ and $k_{2}\left(x,y\right)$ are

(35) $$\begin{array}{cc} \; & {\left.\frac{\partial k_{1}\left(x,y\right)}{\partial y}\right|}_{x=f(r_{0},y),y=0}\\ \; & =\frac{2a_{x}\sqrt{r_{0}^{2}-y_{0}^{2}-z_{0}^{2}}+2a_{z}z_{0}}{vr_{0}}-\frac{vy_{0}^{2}}{r_{0}^{3}}, \end{array}$$

(36) $$\begin{array}{cc} \; & {\left.\frac{\partial k_{2}\left(x,y\right)}{\partial y}\right|}_{x=f(r_{0},y),y=0}\\ \; & =\frac{a_{x}y_{0}}{r_{0}\sqrt{r_{0}^{2}-y_{0}^{2}-z_{0}^{2}}}+\frac{3v^{2}y_{0}^{3}}{2r_{0}^{5}}\\ \; & -\frac{0.5v^{2}y_{0}-3y_{0}\left(a_{x}\sqrt{r_{0}^{2}-y_{0}^{2}-z_{0}^{2}}+a_{z}z_{0}\right)}{r_{0}^{3}}, \end{array}$$

and

(37) $$\begin{array}{cc} {\left.\frac{\partial^{2}k_{2}\left(x,y\right)}{\partial y^{2}}\right|}_{x=f(r_{0},y),y=0}= & \frac{12(a_{x}^{2}+a_{z}^{2})}{v^{2}r_{0}}-\frac{12a_{z}^{2}z_{0}^{2}}{v^{2}r_{0}^{3}}-\frac{15v^{2}y_{0}^{4}}{r_{0}^{7}}-\frac{12a_{x}^{2}\left(r_{0}^{2}-y_{0}^{2}-z_{0}^{2}\right)}{v^{2}r_{0}^{3}}\\ \; & +\frac{36a_{z}y_{0}^{2}z_{0}}{r_{0}^{5}}-\frac{v^{2}}{r_{0}^{3}}-\frac{24a_{x}a_{z}z_{0}{\left(r_{0}^{2}-y_{0}^{2}-z_{0}^{2}\right)}^{0.5}}{v^{2}r_{0}^{3}}\\ \; & -\frac{2a_{x}}{{\left(r_{0}^{2}-y_{0}^{2}-z_{0}^{2}\right)}^{0.5}r_{0}}+\frac{36a_{x}y_{0}^{2}{\left(r_{0}^{2}-y_{0}^{2}-z_{0}^{2}\right)}^{0.5}}{r_{0}^{5}}\\ \; & -\frac{12a_{x}y_{0}^{2}}{{\left(r_{0}^{2}-y_{0}^{2}-z_{0}^{2}\right)}^{0.5}r_{0}^{3}}-\frac{2a_{x}y_{0}^{2}}{{\left(r_{0}^{2}-y_{0}^{2}-z_{0}^{2}\right)}^{1.5}r_{0}}. \end{array}$$

By utilising the Doppler parameter model established above, an azimuth nonlinear chirp scaling (NLCS) [41] is implemented to equalise the translational variant Doppler parameters and get the focused imaging result as follows.

1. Perform a fourth-order filtering processing with respect to range-Doppler domain by multiplying the phase
  (38) $$\begin{array}{c} \phi \left(f_{t}\right)=\exp\left\{j\pi \left(Y_{3}f_{t}^{3}+Y_{4}f_{t}^{4}\right)\right\}. \end{array}$$
2. Equalise the Doppler parameters by multiplying equalisation factor φ(t) with respect to 2 dimensional time domain
  (39) $$\begin{array}{c} \varphi \left(t\right)=\exp\left\{j\pi q_{2}t^{2}+j\pi q_{3}t^{3}+j\pi q_{4}t^{4}\right\}. \end{array}$$
  The expressions of the parameters in the fourth-order filtering and equalisation factors are
  (40) $$\begin{array}{c} \left\{\begin{array}{c} q_{2}=-f_{dc1}\\ q_{3}=\frac{f_{dc1}N}{3{\left(f_{dr0}-f_{dc1}\right)}^{2}}\\ Y_{3}=\frac{f_{dr1}}{3\left(f_{dc1}-f_{dr0}\right)f_{dr0}f_{dc1}}\\ Y_{4}=-\frac{L/6+M(f_{dr0}-f_{dc1})/4}{{\left(f_{dr0}-f_{dc1}\right)}^{3}f_{dr0}^{2}f_{dc1}}\\ q_{4}=\frac{M/4+{\left(f_{dr0}-f_{dc1}\right)}^{3}f_{dr0}^{3}f_{dc1}Y_{4}}{f_{dc1}-f_{dr0}} \end{array}\right.. \end{array}$$
  where
  (41) $$\begin{array}{c} \left\{\begin{array}{c} N=f_{dr1}\left(f_{dr0}-f_{dc1}\right)\\ M=-3Y_{3}f_{dc1}f_{dr1}f_{dr0}^{2}-3q_{3}f_{dr1}\\ L=6q_{3}f_{dr1}\left(f_{dr0}-f_{dc1}\right)\\ +6Y_{3}f_{dc1}f_{dr1}f_{dr2}\left(f_{dr0}f_{dc1}-f_{dc1}^{2}\right)\\ -[f_{dr2}{\left(f_{dr0}-f_{dc1}\right)}^{2}-f_{dr1}^{2}\left(f_{dr0}-f_{dc1}\right)] \end{array}\right.. \end{array}$$
3. Compress the azimuth signal by multiplying azimuth compression factor $\exp\left(\Phi_{az}\left(f_{t}\right)\right)$ in azimuth frequency domain, where $\Phi_{az}\left(f_{t}\right)$ is
  (42) $$\begin{array}{cc} \Phi_{az}\left(f_{t}\right)= & \pi \frac{1}{q_{2}+f_{dr0}}f_{t}^{2}\\ \; & -\pi \frac{Y_{3}f_{dr0}^{3}+q_{3}}{{\left(q_{2}+f_{dr0}\right)}^{3}}f_{t}^{3}\\ \; & -\pi \frac{Y_{4}f_{dr0}^{4}+q_{4}}{{\left(q_{2}+f_{dr0}\right)}^{4}}f_{t}^{4}. \end{array}$$

Afterwards, the imaging result can be obtained, which is discretely represented by z(m,n), where m=1,2,…,M indexes the range time τ and n=1,2,…,N stands for the samples taken along azimuth time *t*, *M* and *N* are the range and azimuth sample numbers, respectively.

Then, we want to use some metric to evaluate the imaging result z(m,n), and formulate the motion parameter estimation problem into an optimisation problem. Lots of image evaluation metrics, including entropy [42,43,44] and sharpness [45,46], have been applied to SAR imaging application. Here, we choose entropy due to its robustness in quality assessment for SAR images. It is generally viewed that a better focusing quality corresponds to a smaller entropy, and therefore the motion parameter estimation can be implemented by finding the global minimum entropy solution, where entropy of the final image is

(43) $$\begin{array}{c} E=\ln S-\frac{1}{S}\sum_{m=0}^{M-1}\sum_{n=0}^{N-1}{\left|z(m,n)\right|}^{2}\ln{\left|z(m,n)\right|}^{2} \end{array}$$

In (43), *S* is the total energy of the imaging result z(m,n),

(44) $$\begin{array}{c} S=\sum_{m=0}^{M-1}\sum_{n=0}^{N-1}{\left|z(m,n)\right|}^{2} \end{array}$$

Then, the optimisation model to estimate the motion parameters $a_{x}$ and $a_{z}$ under the criterion of minimum entropy is

(45) $$\begin{array}{c} \underset{a_{x},a_{z}}{\arg\min}E\left(a_{x},a_{z}\right). \end{array}$$

### 3.5. Motion Estimation Based on Differential Evolution

**Figure 4:**
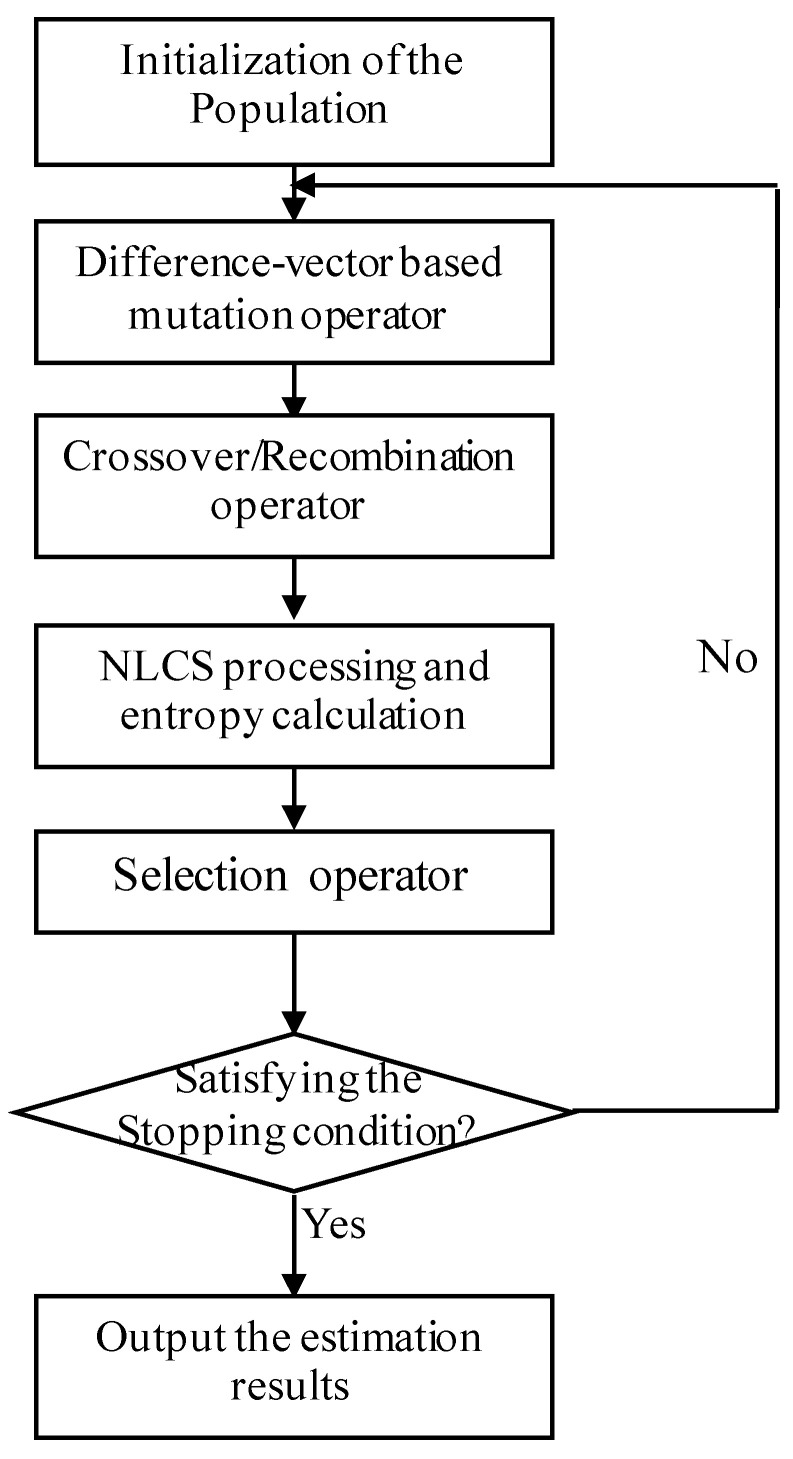
Flowchart of the motion estimation on DE.

In order to search the global minimisation solution to the problem in (45), we use differential evolution (DE) [47,48] optimisation scheme. DE algorithm is an algorithm suitable for solving the global optimal solution in multidimensional space. The algorithm uses differential information of a certain scale among individuals to mutate, a binomial or exponential method to cross, and finally greedy methods to get good individuals into the next generation. The specific procedures to apply DE to solve problem (45) are shown in Figure 4.

In DE algorithm, the global minimisation solution is searched in a *D*-dimensional parameter space, where D=3 for the proposed method. It starts with a randomly initialised *D*-dimensional parameter vector, also known as genome/chromosome. Suppose the subsequent generations of DE are denoted by g=0,1,…,G, and the *i*th vector in the population at *g*th generation is

(46) $$\begin{array}{c} A_{i,g}=\left[a_{x,i,g},a_{z,i,g}\right]. \end{array}$$

As for the acceleration parameters to be estimated here, the values should be limited to a certain range, usually because these parameters are associated with a physical component or with a natural boundary. The initial population (at g=0) should cover this range as much as possible by evenly and randomly allocating individuals within the search space under specified minimum and maximum bounds constraints:

(47) $$\begin{array}{c} \left\{\begin{array}{c} A_{\max}=\left[a_{x\max},a_{z\max}\right]\\ A_{\min}=\left[a_{x\min},a_{z\min}\right] \end{array}\right.. \end{array}$$

Then, the procedures to estimate $a_{x}$ and $a_{z}$ based on the model in (45) are given as follows.

1. **Initialisation of the population:** Randomly initialise the population as
  (48) $$\begin{array}{c} a_{j,i,0}=a_{j\min}+d_{i,j}\left(a_{j\max}-a_{j\min}\right), \end{array}$$
  where j=x or *z*, and $d_{i,j}$ is a random valuable between 0 and 1.
2. **Difference-vector based mutation operator:** Three mutually distinct vectors, $A_{r_{1}^{i},g}$, $A_{r_{2}^{i},g}$ and $A_{r_{3}^{i},g}$, are randomly selected from the population and the donor vector for the *i*th individual is set as
  (49) $$\begin{array}{c} V_{i,g}=A_{r_{1}^{i},g}+F\left(A_{r_{2}^{i},g}-A_{r_{3}^{i},g}\right) \end{array}$$
  where *F* is a scaling factor within [0.4,1].
3. **Crossover/Recombination operator:** This operation uses a recombination operator to exchange donor vector $V_{i,g}$ and the target vector $A_{i,g}$ to generate a test vector $U_{i,g}=\left[u_{x,i,g},u_{y,i,g},u_{z,i,g}\right]$, where
  (50) $$\begin{array}{c} u_{j,i,g}=\left\{\begin{array}{c} v_{j,i,g},\;\mathrm{if}\;\;d_{i,j}\leq C_{r}\;\;\mathrm{or}\;\;j=j_{rand}\\ a_{j,i,g},\;\mathrm{otherwise} \end{array}\right. \end{array}$$
  where $C_{r}$ is a pre-designed crossover factor with [0,1], and $j_{rand}=x$, *y* or *z* is a randomly chosen index ensuring that at least one dimension of test vector $U_{i,g}$ is chosen from the donor vector $V_{i,g}$.
4. **NLCS processing and entropy calculation:** Carry out the NLCS processing and calculate the corresponding image entropy for the imaging result based on the parameter vectors $A_{i,g}$ and $U_{i,g}$, and the entropies corresponding to $A_{i,g}$ and $U_{i,g}$ are denoted as $e_{A}$ and $e_{U}$, respectively.
5. **Selection operator:** According to the requirements of minimising the objective function (image entropy), the next-generation population is selected from the target vector $A_{i,g}$ and the test vector $U_{i,g}$. The specific selection process is as follows.
  (51) $$\begin{array}{c} A_{i,g}=\left\{\begin{array}{c} A_{i,g},\;\mathrm{if}\;\;e_{A}<e_{U}\\ U_{i,g},\;\mathrm{otherwise} \end{array}\right.. \end{array}$$
  Go back to the second step until meeting the stop condition.
6. **Stop condition:** Continue the steps from (2) to (5) until the generation number *g* reaches its maximum value *G*.

## 4. Discussion

In this section, we make several comments about our work:

1. **Motion model:** In this paper, for the simplicity and understandability of the formulas, we only consider accelerations of the radar platform in the motion model. However, in principle, the proposed imaging strategy can be formulated based on a more complex motion model with more higher order terms of the motion trajectory. A more complicated motion model will result in a much more complex derivation of Doppler parameters $f_{dc}$ and $f_{dr}$ in (30) and (31) and a higher-dimensional estimation during DE algorithm.
2. **Imaging processing:** To cope with the translational variant RCM and azimuth modulation in curved trajectory squinted SAR imaging, there have been some proposed imaging algorithms. In this paper, we develop range perturbation [23] and NLCS [41] processes here, which have some approximations and assumptions. We would like to make our effort to combine other more accurate imaging processes—such as Omega-k based algorithm [12,26], NLCS jointly combined with 2-D singular value decomposition (SVD) [25] and time-domain algorithm [17,21]—with our proposed motion modelling and estimation strategy in future work.
3. **Computational cost:** Due to the need of iteratively motion estimation using DE and coarse-to-fine RCM correction scheme, the computational cost of the proposed method is much larger than the existing imaging algorithm for squinted SAR with curved trajectory. However, the proposed method deals with a much more complicated but practical issue—imaging without the accurate motion information of the radar platform. It is a trade-off between complexity of the problem and computational cost. However, with the improvement of computing power, we believe that in the future, computational cost will not be a hindrance to the widespread application of the proposed algorithm.

## 5. Results

In this section, numerical simulations are conducted to evaluate the imaging performance and verify the effectiveness of the proposed method. The imaging performance of the proposed method are further compared with a state-of-the-art method for curved trajectory SAR imaging, i.e., NLCS-based 2-D singular value decomposition (SVD) [25]. Note that the NLCS-based 2-D SVD algorithm is for the case that trajectory is known, and there is no published literature for the case without any information of the movement. Consequently, for the simulation data, the NLCS-based 2-D SVD is implemented with 2 different cases: (1) with accurate movement information and (2) with inaccurate movement information.

It should be stressed that, for the simulated data, our proposed method is implemented without any information of the movement, and we cannot expect it outperforming the NLCS-based 2-D SVD algorithm with accurate movement information. Actually, the similarity of the imaging results between the proposed algorithm and NLCS-based 2-D SVD algorithm with accurate movement information can prove the effectiveness of the proposed method as the proposed method does not use any prior information of the movement. While for the experimental data, the better performance of the proposed method than the NLCS-based 2-D SVD algorithm with INS data can verify the effectiveness of the proposed method.

### 5.1. Experiment on Point-Like Targets

**Figure 5:**
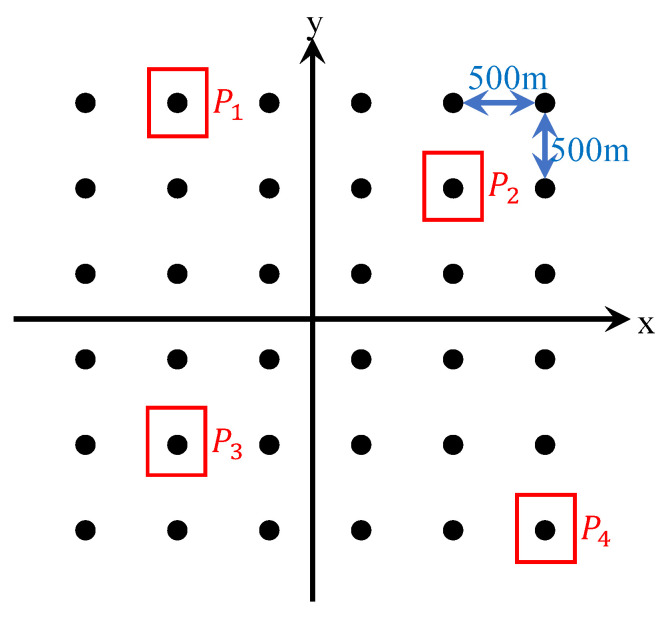
Spatial distribution of point targets.

In the simulation, the imaging algorithms are tested on simulated data of point targets. The spatial distribution of the point targets are illustrated in Figure 5. Thirty-six ideal point targets are evenly distributed in the *x*-*y* plane with 500 m distance between adjacent targets both in *x* and *y* directions. Four point targets, $P_{1}$, $P_{2}$, $P_{3}$ and $P_{4}$, are selected for the presentation in the following.

The corresponding system and geometry parameters for the simulation are listed in Table 1.

Note that during the implementation of the NLCS-based 2-D SVD algorithm with inaccurate movement information, we set the inaccurate accelerations—which are used in the imaging processing—are $2.45\;m/s^{2}$, $1.37\;m/s^{2}$ and $1.88\;m/s^{2}$ with respect to *x*, *y* and *z* directions, respectively. According to the stop condition in (25), the proposed algorithm stops with 12 iterations.

**Figure 6:**
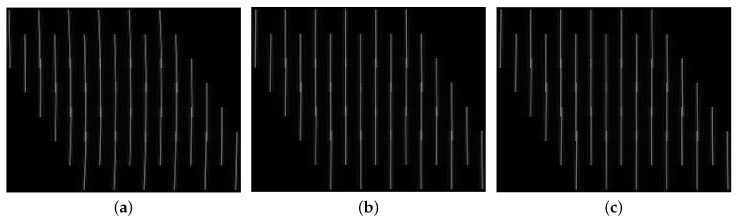
RCM correction results. (**a**) NLCS-based 2-D singular value decomposition (SVD) algorithm with inaccurate movement information. (**b**) NLCS-based 2-D SVD algorithm with accurate movement information. (**c**) The proposed algorithm.

First, in order to show the effectiveness of the proposed algorithm, we show the RCM corrected data of the NLCS-based 2-D SVD algorithm with inaccurate movement information, NLCS-based 2-D SVD algorithm with accurate movement information and the proposed algorithm as shown in Figure 6a–c, respectively.

It can be observed that the RCM trajectories are beyond one range bin for the correction result of NLCS-based 2-D SVD algorithm with inaccurate movement information. Comparatively, RCM can be accurately corrected by the proposed method as the curved RCM trajectories have been changed into straight lines as shown in Figure 6c. The NLCS-based 2-D SVD algorithm with accurate movement information can correct the RCM as well.

**Figure 7:**
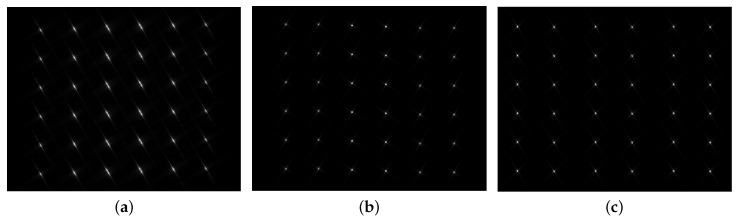
Imaging results. (**a**) Nonlinear chirp scaling (NLCS)-based 2-D SVD algorithm with inaccurate movement information. (**b**) NLCS-based 2-D SVD algorithm with accurate movement information. (**c**) The proposed algorithm.

The imaging results are obtained by exploiting the NLCS-based 2-D SVD algorithm with inaccurate movement information, NLCS-based 2-D SVD algorithm with accurate movement information and the proposed algorithm as shown in Figure 7a–c, respectively. In Figure 7a, the imaging result of NLCS-based 2-D SVD algorithm using inaccurate movement information shows that all the point targets are severely defocused in the presented case. Even a small error occurring the motion parameters will result in a serious degradation on the imaging quality. Compared with Figure 7a, the image qualities in Figure 7b is clearly improved as the accurate movement information is utlized. However, as for the proposed algorithm, we don’t need any prior information of the motion of the radar platform, and we can still get the well-focused imaging result as shown in Figure 7c.

**Figure 8:**
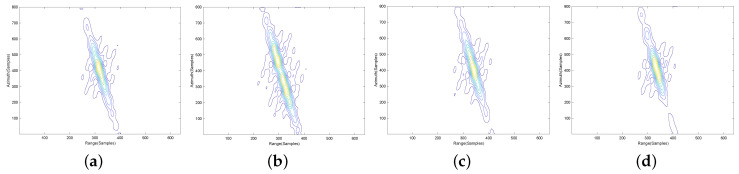
Imaging results of the NLCS-based 2-D SVD algorithm with inaccurate movement information. (**a**) $P_{1}$. (**b**) $P_{2}$. (**c**) $P_{3}$. (**d**) $P_{4}$.

**Figure 9:**
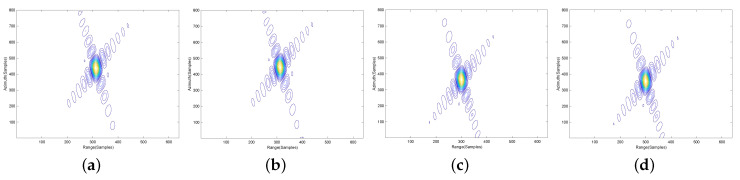
Imaging results of the NLCS-based 2-D SVD algorithm with accurate movement information. (**a**) $P_{1}$. (**b**) $P_{2}$. (**c**) $P_{3}$. (**d**) $P_{4}$.

**Figure 10:**
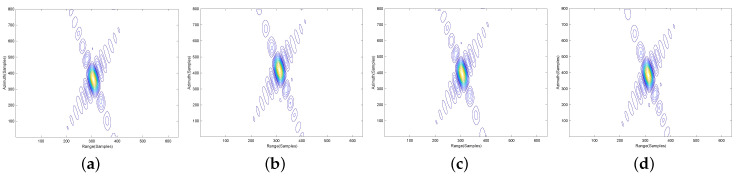
Imaging results of the proposed algorithm. (**a**) $P_{1}$. (**b**) $P_{2}$. (**c**) $P_{3}$. (**d**) $P_{4}$.

To further validate the proposed imaging approach, contour plots of the impulse response function on four different point targets, $P_{1}$, $P_{2}$, $P_{3}$ and $P_{4}$, are shown in the following. To give the details, the results are interpolated eight times. The contours of point targets $P_{1}$, $P_{2}$, $P_{3}$ and $P_{4}$ processed by the NLCS-based 2-D SVD algorithm with inaccurate movement information, NLCS-based 2-D SVD algorithm with accurate movement information and the proposed algorithm are shown in Figure 8, Figure 9 and Figure 10, respectively. Apparently, the NLCS-based 2-D SVD algorithm cannot focus the targets without accurate movement information, but we can have a well-focused results by the proposed method without any prior information of the radar movement.

The quantitative analysis of the measured imaging metrics are given in Table 2 to further evaluate the performance of the proposed method. It should be noted that we do not evaluate the imaging results of the NLCS-based 2-D SVD algorithm with inaccurate movement information as they are obviously unfocused in as shown in Figure 7a and Figure 8. The theoretical values of peak sidelobe ratio (PSLR) and integrated sidelobe ratio (ISLR) are −13.24 dB and −10.23 dB, respectively. The 3 dB impulse response width (IRW) is 1.3 m in the azimuth direction and 1.33 m in the range direction. It can be observed from Table 2 that both of the imaging performance metrics of the proposed algorithm and NLCS-based 2-D SVD algorithm with accurate movement information are close to the theoretical values. The azimuth resolution has a maximum broadening of about 5.4% for the proposed algorithm, and comparatively, it is 3.8% for the NLCS-based 2-D SVD algorithm with accurate movement information. This indicates that the proposed method without movement information can achieve a similar imaging quality as the NLCS-based 2-D SVD algorithm with accurate movement information, which verify the effectiveness of the proposed method.

### 5.2. Experiment on Extended Targets

The results of the proposed technique with state-of-the-art curved trajectory squinted SAR imaging algorithm using SAR data with extended targets are presented in this section. The system and geometry parameters of the experiment is shown in Table 3. Real SAR image is utilised as the extended targets to generate SAR echo.

**Figure 11:**
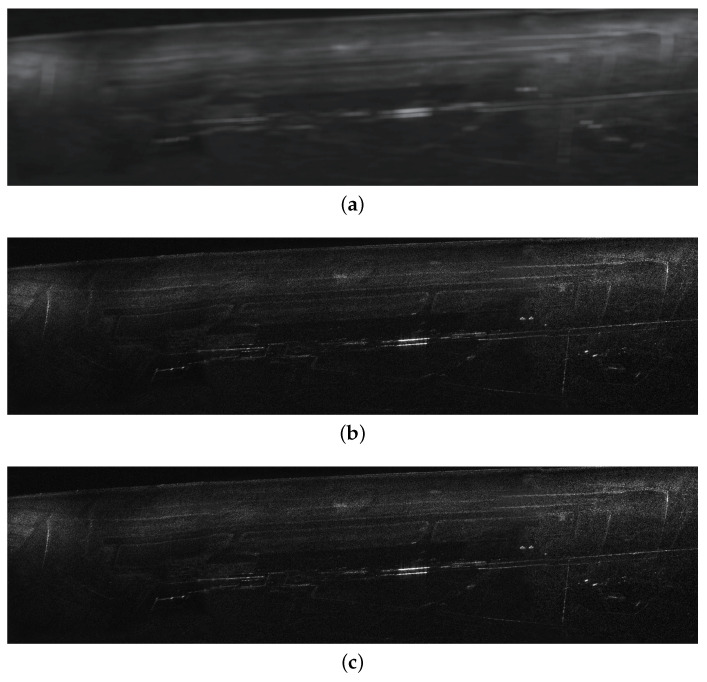
Imaging results of experimental data. (**a**) NLCS-based 2-D SVD algorithm with inaccurate movement information. (**b**) NLCS-based 2-D SVD algorithm with accurate movement information. (**c**) The proposed algorithm.

According to the stop condition in (25), the proposed algorithm stops with 16 iterations. The imaging results of different imaging algorithms are shown in Figure 11, where the image size is 1.8 km × 0.75 km (Range × Azimuth). The horizontal direction is range, and the vertical direction is azimuth. The imaging area is an airport and some neighbouring buildings. The imaging results of NLCS-based 2-D SVD algorithm with inaccurate movement information, NLCS-based 2-D SVD algorithm with accurate movement information and the proposed algorithm are shown in Figure 11a–c, respectively. Note that the in the NLCS-based 2-D SVD algorithm, the motion information of the radar platform is measured from the INS, which is not accurate enough for SAR imaging. In Figure 11a, the imaging result suffers from sever blurring due to the error of the INS data. Compared with Figure 11a, an obvious focusing improvement of the imaging result processed by the proposed algorithm can be observed. The outline of the buildings and roads can be easily recognised. Some strong scatters are perfectly focused with many details being easily identified. The proposed algorithm has a very similar performance and focusing quality as the NLCS-based 2-D SVD algorithm with accurate movement information.

## 6. Conclusions

In terms of the squinted SAR with curved trajectory, this paper makes an attempt of imaging the SAR echo without any movement information of the radar platform. A novel motion modelling and estimation algorithm is proposed to implement SAR imaging and motion information estimation at the same time. In the proposed method, range perturbation and NLCS processes are utilised to solve the problems stemming from the translational variant RCM and Doppler parameters, while DE algorithm is applied to estimate the motion parameters. We empirically compare the proposed algorithms with state-of-the-art imaging algorithms for SAR with curved trajectory on synthetic SAR data, and the results verify the effectiveness of the proposed method.

Some future challenges on our work lie in the following aspects.

- In order to improve the image quality further, we plan to conduct some more accurate imaging processes with our proposed motion modelling and estimation strategy in future work.
- In order to reduce the computational cost of the proposed algorithm, the authors would like to develop some other efficient global optimisation algorithm to replace DE during the estimation of motion parameters.

## Figures and Tables

**Table 1:** Simulation parameters.

Parameter	Value
$f_{c}$: Carrier frequency	9.6GHz
$B_{r}$: Signal bandwidth	100MHz
$T_{r}$: Signal time width	1.5¯s
$f_{s}$: Range time sampling rate	150MHz
$T_{s}$: Synthetic aperture time	0.7s
*v*: Radar platform velocity	86 m/s
PRF: Pulse repetition frequency	200Hz
$x_{0}$: Radar position in *x* direction at initial time	−1km
$y_{0}$: Radar position in *y* direction at initial time	−3km
$z_{0}$: Radar position in *z* direction at initial time	2km
$a_{x}$: acceleration in *x* direction	$2.5\;m/s^{2}$
$a_{z}$: acceleration in *z* direction	$1.9\;m/s^{2}$

**Table 2:** Imaging quality evaluation.

		Azimuth			Range		
		PSLR(dB)	ISLR(dB)	IRW(m)	PSLR(dB)	ISLR(dB)	IRW(m)
Proposed method	*P*_1_	−13.20	−10.19	1.35	−13.19	−10.22	1.35
	*P*_2_	−13.23	−10.23	1.31	−13.22	−10.20	1.34
	*P*_3_	−13.22	−10.22	1.32	−13.24	−10.23	1.33
	*P*_4_	−13.14	−10.15	1.37	−13.17	−10.17	1.36
NlCS based 2-D SVD algorithm (with accurate movement information)	*P*_1_	−13.24	−10.22	1.33	−13.23	−10.22	1.34
	*P*_2_	−13.23	−10.23	1.31	−13.24	−10.23	1.33
	*P*_3_	−13.24	−10.23	1.30	−13.24	−10.24	1.33
	*P*_4_	−13.22	−10.21	1.35	−13.22	−10.22	1.35

**Table 3:** Experimental parameters.

Parameter	Value
$f_{c}$: Carrier frequency	9.6GHz
$B_{r}$: Signal bandwidth	300MHz
$T_{r}$: Signal time width	1.5¯s
$f_{s}$: Range time sampling rate	400MHz
$T_{s}$: Synthetic aperture time	0.7s
*v*: Radar platform velocity	147 m/s
PRF: Pulse repetition frequency	1000Hz
$x_{0}$: Radar position in *x* direction at initial time	−0.8km
$y_{0}$: Radar position in *y* direction at initial time	−1.6km
$z_{0}$: Radar position in *z* direction at initial time	1.3km

## References

[1] Reigber A., Scheiber R., Jager M., Prats-Iraola P., Hajnsek I., Jagdhuber T., Papathanassiou K.P., Nannini M., Aguilera E., Baumgartner S. (2013). Very-high-resolution airborne synthetic aperture radar imaging: Signal processing and applications. Proc. IEEE.

[2] Pu W., Wu J. (2020). OSRanP: A Novel Way for Radar Imaging Utilizing Joint Sparsity and Low-Rankness. IEEE Trans. Comp. Imag..

[3] Moreira A., Prats-Iraola P., Younis M., Krieger G., Hajnsek I., Papathanassiou K.P. (2013). A tutorial on synthetic aperture radar. IEEE Geosci. Remote Sens. Mag..

[4] Hu R., Li X., Yeo T.S., Yang Y., Chi C., Zuo F., Hu X., Pi Y. (2019). Refocusing and Zoom-In Polar Format Algorithm for Curvilinear Spotlight SAR Imaging on Arbitrary Region of Interest. IEEE Trans. Geosci. Remote Sens..

[5] Cantalloube H.M.J., Nahum C.E. (2013). Airborne SAR-efficient signal processing for very high resolution. Proc. IEEE.

[6] Pu W., Wang X., Wu J., Huang Y., Yang J. (2020). Video SAR Imaging Based on Low-Rank Tensor Recovery. IEEE Trans. Neural Netw. Learn. Syst..

[7] Xing M., Wu Y., Zhang Y.D., Sun G., Bao Z. (2014). Azimuth Resampling Processing for Highly Squinted Synthetic Aperture Radar Imaging With Several Modes. IEEE Trans. Geosci. Remote Sens..

[8] Bi H., Wang J., Bi G. (2019). Wavenumber Domain Algorithm-Based FMCW SAR Sparse Imaging. IEEE Trans. Geosci. Remote Sens..

[9] Bie B., Sun G., Xia X., Xing M., Guo L., Bao Z. (2019). High-Speed Maneuvering Platforms Squint Beam-Steering SAR Imaging Without Subaperture. IEEE Trans. Geosci. Remote Sens..

[10] Ran L., Xie R., Liu Z., Zhang L., Li T., Wang J. (2018). Simultaneous Range and Cross-Range Variant Phase Error Estimation and Compensation for Highly Squinted SAR Imaging. IEEE Trans. Geosci. Remote Sens..

[11] Meng Z., Li Y., Li C., Xing M., Bao Z. (2015). A raw data simulator for Bistatic Forward-looking High-speed Maneuvering-platform SAR. Signal Process..

[12] Li Z., Liang Y., Xing M., Huai Y., Gao Y., Zeng L., Bao Z. (2016). An Improved Range Model and Omega-K-Based Imaging Algorithm for High-Squint SAR with Curved Trajectory and Constant Acceleration. IEEE Geosci. Remote Sens. Lett..

[13] Li Y., Song X., Guo L., Mei H., Quan Y. (2020). Inverse-mapping filtering Polar Formation Algorithm for High-maneuverability SAR with time-variant acceleration. Signal Process..

[14] Liao Y., Zhou S., Yang L. (2018). Focusing of SAR with Curved Trajectory Based on Improved Hyperbolic Range Equation. IEEE Geosci. Remote Sens. Lett..

[15] Vu V.T., Pettersson M.I. (2015). Nyquist sampling requirements for polar grids in bistatic time-domain algorithms. IEEE Trans. Signal Process..

[16] Rodriguez-Cassola M., Prats P., Krieger G., Moreira A. (2011). Efficient time-domain image formation with precise topography accommodation for general bistatic SAR configurations. IEEE Trans. Aerosp. Electron. Syst..

[17] Xie H., An D., Huang X., Zhou Z. (2015). Fast Time-Domain Imaging in Elliptical Polar Coordinate for General Bistatic VHF/UHF Ultra-Wideband SAR With Arbitrary Motion. IEEE J. Sel. Top. Appl. Earth Observ. Remote Sens..

[18] Feng D., An D., Huang X. (2018). An Extended Fast Factorized Back Projection Algorithm for Missile-Borne Bistatic Forward-Looking SAR Imaging. IEEE Trans. Aerosp. Electron. Syst..

[19] Dong Q., Sun G., Yang Z., Guo L., Xing M. (2018). Cartesian Factorized Backprojection Algorithm for High-Resolution Spotlight SAR Imaging. IEEE Sens. J..

[20] Ran L., Liu Z., Li T., Xie R., Zhang L. (2018). An Adaptive Fast Factorized Back-Projection Algorithm with Integrated Target Detection Technique for High-Resolution and High-Squint Spotlight SAR Imagery. IEEE J. Sel. Top. Appl. Earth Observ. Remote Sens..

[21] Pu W., Wang X., Huang Y., Yang J. (2019). Fast Factorized Back Projection Imaging Algorithm integrated with Motion Error Estimation for Bistatic Forward-looking SAR. IEEE J. Sel. Top. Appl. Earth Observ. Remote Sens..

[22] Wu J., Li Y., Pu W., Li Z., Yang J. (2019). An Effective Autofocus Method for Fast Factorized Back-Projection. IEEE Trans. Geosci. Remote Sens..

[23] Dang Y., Liang Y., Bie B., Ding J., Zhang Y. (2018). A Range Perturbation Approach for Correcting Spatially Variant Range Envelope in Diving Highly Squinted SAR With Nonlinear Trajectory. IEEE Geosci. Remote Sens. Lett..

[24] Chen S., Zhao H., Zhang S., Chen Y. (2014). An extended nonlinear chirp scaling algorithm for missile borne SAR imaging. Signal Process..

[25] Chen J., Sun G., Xing M., Liang B., Gao Y. (2019). Focusing Improvement of Curved Trajectory Spaceborne SAR Based on Optimal LRWC Preprocessing and 2-D Singular Value Decomposition. IEEE Trans. Geosci. Remote Sens..

[26] Li Z., Xing M., Xing W., Liang Y., Gao Y., Dai B., Hu L., Bao Z. (2017). A Modified Equivalent Range Model and Wavenumber-Domain Imaging Approach for High-Resolution-High-Squint SAR with Curved Trajectory. IEEE Trans. Geosci. Remote Sens..

[27] Pu W., Wu J., Huang Y., Yang J., Li W., Yang H. (2019). Joint Sparsity-based Imaging and Motion Error Estimation for BFSAR. IEEE Trans. Geosci. Remote Sens..

[28] Xing M., Jiang X., Wu R., Zhou F., Bao Z. (2009). Motion compensation for UAV SAR based on raw radar data. IEEE Trans. Geosci. Remote Sens..

[29] Hu R., Rao B.S., Alaee-Kerahroodi M., Ottersten B. (2020). Orthorectified Polar Format Algorithm for Generalized Spotlight SAR Imaging With DEM. IEEE Trans. Geosci. Remote Sens..

[30] Zhang L., Qiao Z., Xing M.-D., Yang L., Bao Z. (2012). A robust motion compensation approach for UAV SAR imagery. IEEE Trans. Geosci. Remote Sens..

[31] Pu W., Wu J., Huang Y., Li W., Sun Z., Yang J., Yang H. (2016). Motion Errors and Compensation for Bistatic Forward-Looking SAR With Cubic-Order Processing. IEEE Trans. Geosci. Remote Sens..

[32] Wahl D.E., Eichel P., Ghiglia D.C., Jakowatz C.V. (1994). Phase gradient autofocus: A robust tool for high resolution SAR phase correction. IEEE Trans. Aerosp. Electron. Syst..

[33] Pu W., Wu J., Huang Y., Yang J., Li W., Yang H. (2017). A Rise-dimensional Modeling and Estimation method for Flight Trajectory Error in Bistatic Forward-looking SAR. IIEEE J. Sel. Top. Appl. Earth Observ. Remote Sens..

[34] Samczynski P., Kulpa K.S. (2010). Coherent mapdrift technique. IEEE Trans. Geosci. Remote Sens..

[35] Zhao S., Deng Y., Wang R. (2017). Imaging for High-Resolution Wide-Swath Spaceborne SAR Using Cubic Filtering and NUFFT Based on Circular Orbit Approximation. IEEE Trans. Geosci. Remote Sens..

[36] Pu W., Huang Y., Wu J., Yang J., Li W., Yang H. A minimum-entropy based residual range cell migration correction for bistatic forward-looking SAR. Proceedings of the 2016 IEEE Radar Conference (RadarConf).

[37] Pu W., Wu J., Huang Y., Yang J., Li W., Yang H. (2018). Nonsystematic Range Cell Migration (NsRCM) Analysis and Autofocus Correction for Bistatic Forward-looking SAR. IEEE Trans. Geosci. Remote Sens..

[38] Mao X., Zhu D., Zhu Z. (2013). Autofocus correction of APE and residual RCM in spotlight SAR polar format imagery. IEEE Trans. Aerosp. Electron. Syst..

[39] Zeng L., Liang Y., Xing M., Li Z., Huai Y. (2016). Two-dimensional autofocus technique for high-resolution spotlight synthetic aperture radar. IET Signal Process..

[40] Zhong H., Zhang Y., Chang Y., Liu E., Tang X., Zhang J. (2018). Focus High-Resolution Highly Squint SAR Data Using Azimuth-Variant Residual RCMC and Extended Nonlinear Chirp Scaling Based on a New Circle Model. IEEE Geosci. Remote Sens. Lett..

[41] Sun Z., Wu J., Li Z., Huang Y., Yang J. (2015). Highly Squint SAR Data Focusing Based on Keystone Transform and Azimuth Extended Nonlinear Chirp Scaling. IEEE Geosci. Remote Sens. Lett..

[42] Xiong T., Xing M., Wang Y., Wang S., Sheng J., Guo L. (2014). Minimum-entropy-based autofocus algorithm for SAR data using chebyshev approximation and method of series reversion, and its implementation in a data processor. IEEE Trans. Geosci. Remote Sens..

[43] Zeng T., Wang R., Li F. (2013). SAR Image Autofocus Utilizing Minimum-Entropy Criterion. IEEE Geosci. Remote Sens. Lett..

[44] Wang J., Liu X. (2006). SAR Minimum-Entropy Autofocus Using an Adaptive-Order Polynomial Model. IEEE Geosci. Remote Sens. Lett..

[45] Morrison R.L., Do M.N., Munson D.C. (2007). SAR image autofocus by sharpness optimization: A theoretical study. IEEE Trans. Image Process..

[46] Gao Y., Yu W., Liu Y., Wang R., Shi C. (2014). Sharpness-based autofocusing for stripmap SAR using an adaptive-order polynomial model. IEEE Trans. Geosci. Remote Sens..

[47] Das S., Suganthan P.N. (2011). Differential evolution: A survey of the state-of-the-art. IEEE Trans. Evol. Comput..

[48] Storn R., Price K. (1995). Differential Evolution—A Simple and Efficient Adaptive Scheme for Global Optimization over Continuous Spaces.

